# Morphology and Intrinsic Excitability of Regenerating Sensory and Motor Neurons Grown on a Line Micropattern

**DOI:** 10.1371/journal.pone.0110687

**Published:** 2014-10-20

**Authors:** Ouafa Benzina, Thierry Cloitre, Marta Martin, Cédric Raoul, Csilla Gergely, Frédérique Scamps

**Affiliations:** 1 Institut National de la Santé et de la Recherche Médicale, Inserm UMR1051, the neuroscience institute of Montpellier, Saint Eloi hospital, Montpellier, France; 2 Université Montpellier 2, Laboratoire Charles Coulomb UMR 5221, Montpellier, France; 3 CNRS, Laboratoire Charles Coulomb UMR 5221, Montpellier, France; 4 Université Montpellier 1, 2, Montpellier, France; University of Milan-Bicocca, Italy

## Abstract

Axonal regeneration is one of the greatest challenges in severe injuries of peripheral nerve. To provide the bridge needed for regeneration, biological or synthetic tubular nerve constructs with aligned architecture have been developed. A key point for improving axonal regeneration is assessing the effects of substrate geometry on neuronal behavior. In the present study, we used an extracellular matrix-micropatterned substrate comprising 3 µm wide lines aimed to physically mimic the *in vivo* longitudinal axonal growth of mice peripheral sensory and motor neurons. Adult sensory neurons or embryonic motoneurons were seeded and processed for morphological and electrical activity analyses after two days *in vitro*. We show that micropattern-guided sensory neurons grow one or two axons without secondary branching. Motoneurons polarity was kept on micropattern with a long axon and small dendrites. The micro-patterned substrate maintains the growth promoting effects of conditioning injury and demonstrates, for the first time, that neurite initiation and extension could be differentially regulated by conditioning injury among DRG sensory neuron subpopulations. The micro-patterned substrate impacts the excitability of sensory neurons and promotes the apparition of firing action potentials characteristic for a subclass of mechanosensitive neurons. The line pattern is quite relevant for assessing the regenerative and developmental growth of sensory and motoneurons and offers a unique model for the analysis of the impact of geometry on the expression and the activity of mechanosensitive channels in DRG sensory neurons.

## Introduction

Following peripheral nerve injury, spinal motoneurons and dorsal root ganglia (DRG) sensory neurons have to adapt to a new environment to successfully promote axonal elongation. Unsuccessful regeneration leads to palsy, ataxia and in the occurrence and maintenance of pain-related behavior. Understanding the cellular and molecular mechanisms leading to improved neurite re-growth is a major step to propose new therapies for sensory-motor nerve repair.

It has been demonstrated for many years that prior lesion of the peripheral nerve (conditioning lesion) leads to more rapid sensory and motor recovery following a second nerve injury [1]–[3]. The conditioning peripheral nerve lesion converts the arborizing mode of growth of adult sensory neurons commonly observed *in vitro* into an elongating mode characterized by longer neurite length, reduced branching and faster growth velocity [4]–[6]. This experimental paradigm represents therefore an attractive means to identify factors improving regeneration. In addition to morphological changes, the conditioning lesion increased neuronal excitability in a subset of sensory neurons, which could contribute to neuropathic pain [7], [8]. These *in vitro* experiments led to large scale *in vivo* transcriptional analysis of genes involved into the intrinsic growth capacity of sensory and motoneurons [9] that, together with the activation of environmental factors allows the regenerative process [10]. Moreover, we demonstrated that conditioned sensory neurons displayed different rheological properties, including a decrease in soma and growth cones membrane elasticity [11], [12]. These last data highlighted that, in addition to chemical signals, physical parameters had to be considered in neurite outgrowth process.

The structural organization of tissues plays a major role in influencing degree and direction of tissue growth. *In vivo*, the peripheral nerve growth is guided longitudinally along a basal lamina within the Schwann cell bands (or tubes). The directional axonal elongation observed following an injury, is mainly based on the interactions between regenerating axons and the adjacent substratum [13]. Numerous studies have assessed the effects of synthetic micro- and nano-architectures to provide a structural support for axonal regrowth under a large loss of matter [14]–[17]. This approach fulfills biomimetic considerations by mimicking *in vivo* cell architecture and physiology. However, it is now established that cell behavior can be greatly influenced by topographical signals [14], [18]–[20]. Therefore, assessing the effects of substrate geometry on neuronal behavior is an important point for understanding axonal regeneration. In the present study, we used an extracellular matrix-micropatterned substrate aimed to physically mimic *in vivo* longitudinal axonal growth. We used this pattern to decipher the role of cell geometry on sensory and motoneurons neurite growth and electrical properties under different pathophysiological conditions.

## Results

### Effect of topographical cues on neurite growth of DRG sensory neurons

**Figure 1:**
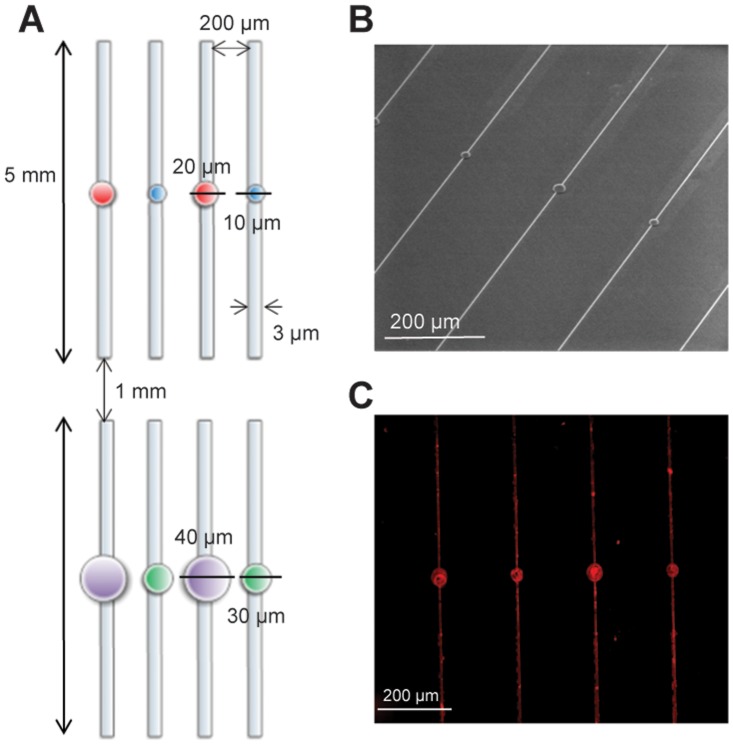
Micropattern preparation. (A) schematic diagram of the pattern, reproduced on a 1 cm^2^ total surface. The designed patterns consist in 3 µm width and 5 mm long parallel lines separated by a distance of 200 µm to avoid cross-talk between each other. In addition, each line contains in the middle a microplot of various, 10, 20, 30 or 40 µm diameters. This pattern is printed on a chromium mask that is used for fabrication of a silicon mold. (B) electron microscopy image of PDMS stamp after photolithography, (C) immunofluorescence image obtained using anti-laminin antibody to visualize the laminin coated patterns (lines and plots).

**Figure 2:**
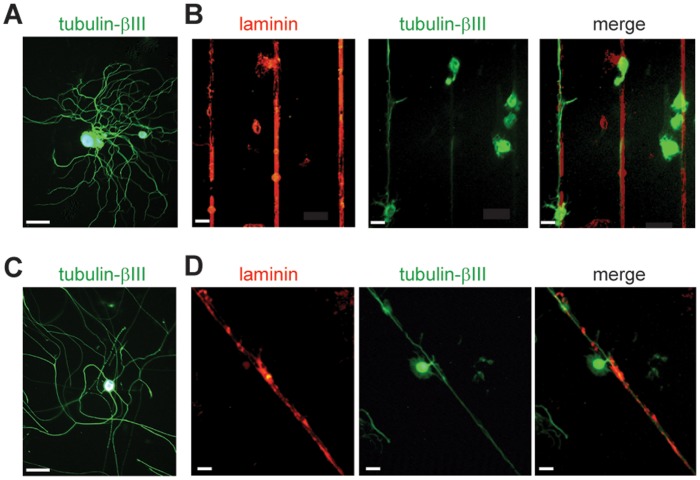
Immunolabeling of sensory neurons at 2 DIV with βIII tubulin antibody. (A) control neuron grown on unpatterned laminin-coated coverslips extend branched neurites, (B) control neuron adhere onto the lines of ECM patterned substrate and extend one or two elongated neurites (red anti-laminin antibody allows to visualize lines; and green anti-βIII tubulin antibody stains neurites and soma of neurons). (C) Conditioned neurons grown on unpatterned laminin-coated coverslips extend elongated neurites. (D) Conditioned neurons extend one or two elongated neurites on patterned substrate. (Scale bar, 50 µm).

**Figure 3:**
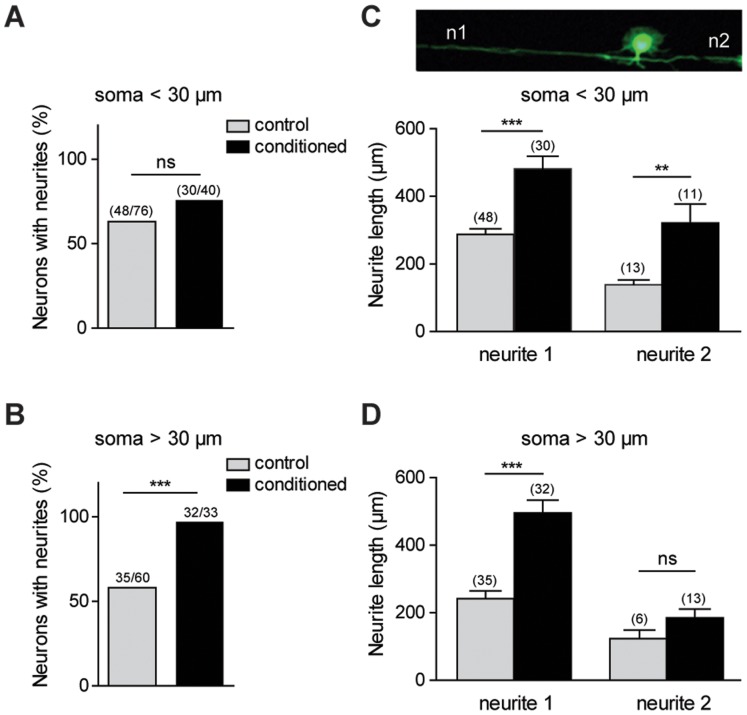
Quantification of neurite length of control and conditioned neurons growing on micropatterned substrate. (A, C) analysis of patterned small somatic size neurons shows that conditioning does not increase the number of neurons extending a neurite, but increased maximal neurite length. (B, D) analysis of patterned large somatic size neurons shows that conditioning both increases the number of neurons extending a neurite and the maximal neurite length. (C and D **p<0.01, ***p<0.001, *t* test; A and B ***p<0.001, Chi-square (and Fisher’s exact), *n* = 3 cultures).

Sensory neurons were seeded on the micropattern composed of parallel lines and microplots as described in Methods (Figure 1A). Figure 1B presents the electron microscopy image of the obtained PDMS stamps after photolithography. The printed features were visualized using immunofluorescence with an anti-laminin antibody staining (Figure 1C). After two days *in vitro*, cells were fixed and immunostained to evaluate the effects of microplots and line guidance on adhesion and neurite growth of control (contralateral DRG) and conditioned (ipsilateral DRG) adult sensory neurons. The use of different plot sizes was aimed to promote differential adhesion of sub-populations of sensory neurons. DRG sensory neurons form a morphologically and functionally heterogeneous population coding for different modalities such as pain, touch, temperature and proprioception [21]. A somatic size criterion is commonly used in *in vitro* electrophysiological analyses to roughly select the small-size nociceptive neuronal population from the innocuous population [22]–[24]. The use of specific molecular markers confirmed that the small somatic diameter neurons (<30 µm) are the pain and temperature-sensing neurons. The medium-large somatic diameter neurons (>30 µm) comprise the proprioceptors, the innocuous and probably a subset of painful mechanoceptors [25]. Regarding neuron somatic size, we did not observe any adhesive selectivity between plots and lines. Henceforth, analyses were performed on patterned neurons independently of their position relative to plots or lines. In a recent study, we shown no differences in soma size distribution between control and conditioned neurons, except a loss of largest size neurons after axotomy [11]. Although the neurite width is three-fold less than the line width (3 µm), control or conditioned sensory neurons grew only one or two neurites, without secondary branching, on our line micro patterned substrate (Figure 2B, D), which is rarely observed on unpatterned substrate (Figure 2A, C). Whatever their somatic size, approximately 60% of sensory neurons grew at least one neurite after 2 days *in vitro* (DIV) (Figure 3A, B). Following prior nerve injury *in vivo*, the percentage of neurons having at least one neurite significantly increased among the large population of sensory neurons reaching nearly 100% (Figure 3A, B). Analysis of the number of neurites show that conditioning nerve injury does not promote the growth of a second neurite among small size neurons 27% (13/48) and 36% (11/30) and large size neurons 17% (6/35) and 40% (13/32), in control and conditioned neurons, respectively (*p*>0.05, unpaired two-tailed *t* test). Analysis of neurite length shows that conditioning injury promotes longer neurites on a patterned substrate in all sensory neurons populations (Figure 3C, D), a result in agreement with previous results reported on unpatterned substrate [4]. However, an increase in the length of the second neurite was observed only among the small size neurons (Figure 3C, D). Whatever the neuronal population or experimental condition, length of the second neurite was always shorter. Therefore, 3-µm line geometry is sufficient to promote neuron adhesion, forces neurite growth toward an elongated mode and preserves the stimulating effects of conditioning on neurite length.

### Effect of patterned substrate on cellular diversity and electrical properties of large somatic size sensory neurons

**Figure 4:**
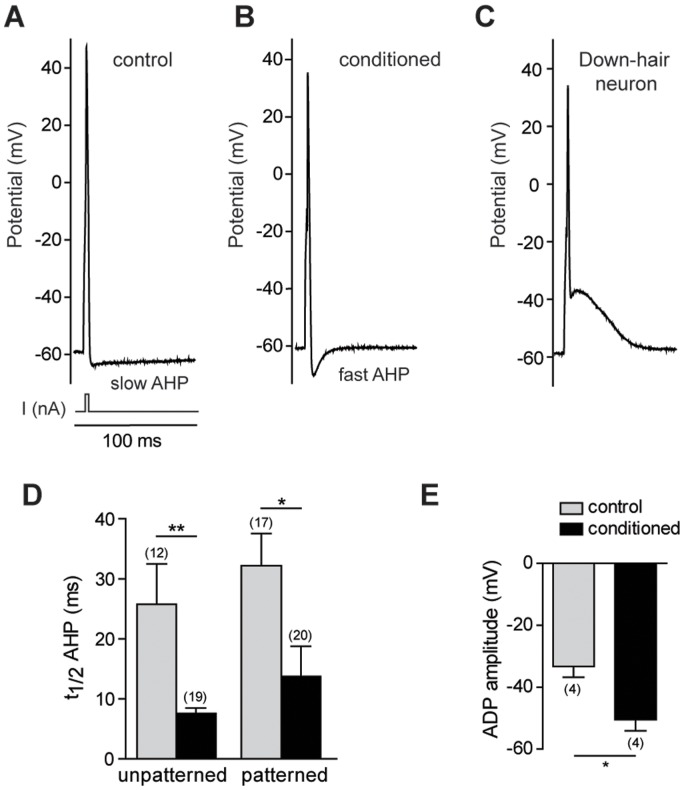
Effects of pattern on electrical activity of large sensory neurons. Typical action potential recordings elicited with a 2 ms depolarizing current injection and showing either slow (A) or fast (B) afterhyperpolarization (AHP). (C) Random electrophysiological recordings of patterned sensory neurons evidenced the presence of an afterdepolarization (ADP), characteristic of a subset of sensory neurons, the Down-hair mechanoreceptors. (D) Conditioned sensory neurons preferentially express a fast AHP, measured as the half time for baseline recovery from the peak hyperpolarization amplitude. (E) The pattern does not prevent the decrease in the maximal amplitude of the ADP in conditioned neurons (*p<0.05, Mann Whitney test).

We previously showed that, in addition to promoting neurite growth, a conditioning nerve injury modified the electrical properties of large sized sensory neurons [8]. As cell morphology can affect excitability [26], we aimed to determine the effects of patterning on electrical activity of control and conditioned sensory neurons having a somatic size superior to 30 µm. All patterned adult DRG sensory neurons recorded at 2 DIV were able to fire an action potential under injection of a short depolarizing current. Following an action potential, two types of hyperpolarization (AHP) were observed, a slow AHP (Figure 4A) and a fast AHP (Figure 4B). A subset of neurons displayed an after depolarization (ADP) (Figure 4C), previously identified as being characteristic to a sub-type of mechanoreceptor innervating the hair follicles, the Down-hair mechanoreceptors [22]. We found that the fast AHP was a hallmark of conditioned neurons, while the slow AHP characterized control neurons (Figure 4D). The percentage of neurons with an ADP was also maintained on line patterning (approximately 20% of large neurons) and the amplitude of ADP decreased following lesion-conditioning (Figure 4E) as previously reported [27]. Further analysis of intrinsic electrical properties of DRG sensory neurons expressing an AHP (80% of large sensory neurons) evidenced that control neurons tended to have a larger action potential duration that was exacerbated on patterned substrate (Table 1). Overall our data indicate that line micropatterning maintains the cellular diversity of DRG sensory neurons under pathophysiological conditions.

**Table 1:** Action potential characteristics of control and conditioned sensory neurons.

		Unpatterned ECM			Patterned ECM	
	RMP (mV)	AP peak (mV)	AP half width (ms)	RMP (mV)	AP peak (mV)	AP half width (ms)
Control	−61.6	37.8^a^*	0.95^a^*	−60.2	29.1	1.4^b^***
Conditioned	−60.2	37.6	0.78	−60.7	30.5	0.69

a, compared with control patterned; b, compared with patterned conditioned. *p<0.5, ***p<0.001, unpaired t-test. RMP, resting membrane potential; AP, action potential.

### Effect of patterned substrate on sensory neurons excitability

**Figure 5:**
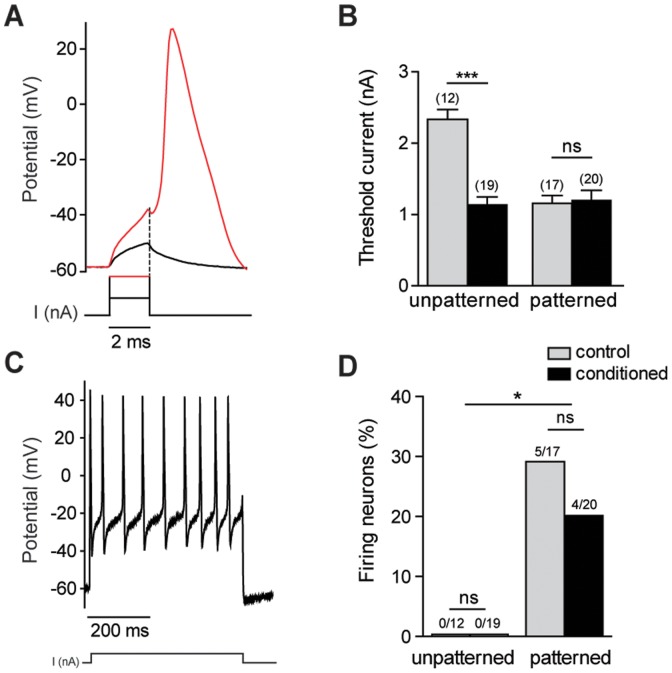
Effects of pattern on excitability of large sensory neurons. (A) Typical recordings of an action potential elicited by threshold current (all or nothing response). Black lines: 2 ms current amplitude does not trigger an AP. Red lines: increasing current amplitude triggers a full AP. (B) Unlike unpatterned substrate, the amplitude of current that triggers an action potential is not significantly different between control and axotomized sensory neurons grown on patterned substrate (***p<0.001, *t* test). (C) Typical firing action potentials of patterned neurons induced by long depolarizing current (500 ms duration). (D) Maintained firing was recorded in roughly 20–30% of patterned control and conditioned neurons. No firing activity was observed on unpatterned neurons recorded at 2 DIV. (*p<0.05, Chi-square, Fisher test).

Neuronal excitability was assessed by measuring the minimal current amplitude required to trigger an action potential, the threshold current (Figure 5A–B) and by determining the propensity of neurons to fire several action potentials in response to a long lasting current (Figure 5C–D). No significant differences in threshold current were found between patterned control and conditioned neurons (Figure 5B). However, patterning induced a significant decrease in the threshold current amplitude for control neurons (1.1±0.1 nA, *n* = 17) compared with control unpatterned neurons (2.3±0.1 nA, *n* = 12, *p*<0.001, unpaired two-tailed *t* test). Among conditioned neurons, no significant differences in the threshold current were observed between patterned (1.2±0.1 nA, *n* = 20) and unpatterned neurons (1.1±0.1 nA, *n* = 19). Remarkably, line pattern revealed a subset of control and conditioned neurons (20–30%) able to fire continuously action potentials under a maintained current application, an electrical behaviour consistent with the functional properties of subclasses of mechano-proprioceptors (Figure 5D)[28]. These data demonstrate that a line micropatterned substrate impacts the excitability of sensory neurons.

### Effect of patterned substrate on neurite polarity

**Figure 6:**
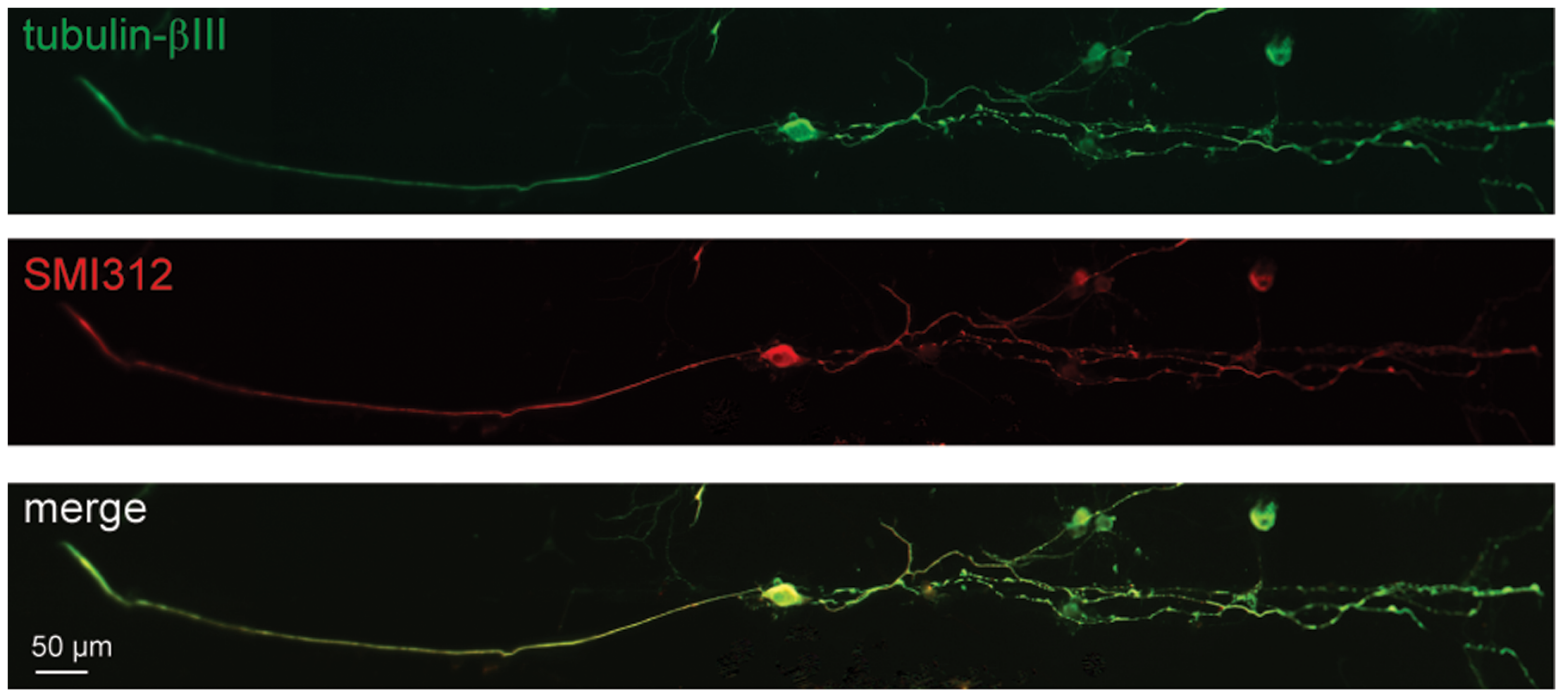
Immunostaining of patterned sensory neuron polarity. Image of double immunostaining with the neuronal cytoskeletal marker anti-βIII tubulin (green) and axonal marker anti-SMI312 (red) shows that both neurites expressed the axonal marker (merge image).

**Figure 7:**
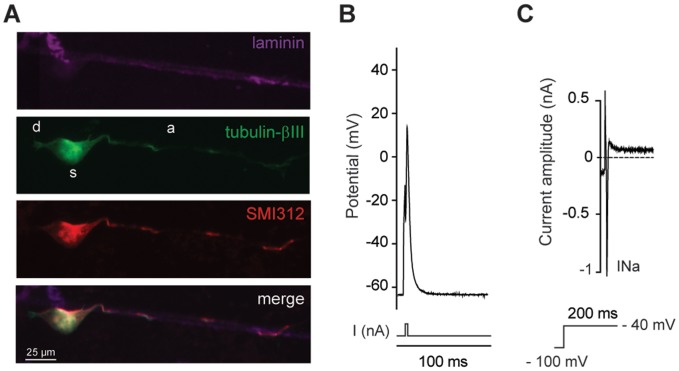
Effects of pattern on spinal motoneurons polarity and electrical properties. (A) Image of triple staining of a patterned *Hb9::GFP* embryonic motoneuron with anti-laminin (pink) to reveal the pattern, anti-GFP (green) to enhance motoneuron morphology and the axonal marker anti-SMI312 (red). Motoneuron shows polarity with one long axon (red) and short dendrites (d, green). Hb9^+^-GFP motoneurons were fixed at 2 DIV (d, dendrite; s, soma and a, axon). (B) Patterned embryonic motoneurons begin to express electrical activity at 2DIV following the injection of 2 ms current. (C) Voltage clamp recordings on the same neuron evidenced the expression of sodium current (INa). Voltage protocol is shown below current trace.

DRG sensory neurons are called unipolar or T-neurons due to the emergence of two axonal branches from one common nerve extension. The peripheral branch receives inputs from skin, muscles, joints, and viscera, but is considered as an axon, not a dendrite. The central branch projects into the spinal cord and is an axon. The line pattern imposes sensory neurons to grow one or two neurites, which led us to investigate whether a polarity exists between an axonal and a “dendrite” like growth. To differentiate between axon and dendrite, we co-stained sensory neurons with pan-axonal neurofilament marker SMI312 and a cytoskeletal neuronal marker βIII-tubulin. We found that SMI312 immunostained both neurites in most large size DRG sensory neurons, suggesting these neurons extend two axons (Figure 6). Interestingly, numerous small size neurons were negative to SMI312, which supports that DRG sensory neurons with SMI312 immunostained process might belong to the subpopulation that becomes myelinated *in vivo*, as reported for the neurofilament 200 kDa [29]. This subpopulation specific SMI312 staining was also observed on unpatterned substrate independently of neurite numbers (Figure S1). To reinforce that neuronal polarity and identity is maintained on the line micropatterned substrate, we analyzed process growth of central neurons, the spinal motoneurons whose axons constitute the mixed sensory-motor peripheral nerve. Consistent with polarity of central neurons, patterned *Hb9::GFP* motoneurons extended a long SMI312-positive axon at 2 DIV and small SMI312-negative dendrites (Figure 7A). To confirm the specificity of SMI312 axonal staining, we show that the dendrite specific marker, microtubule-associated protein 2 (Map2), stained small neurites, not the long axon of motoneurons at 6 DIV (Figure S2). We observed that patterned embryonic motoneurons have an electrical activity after 2 DIV (Figure 7B) according to the presence of fast sodium current (Figure 7C).

## Discussion

We here evaluated whether a structural guidance based on adhesive micropattern keeps polarization of neurite growth of sensory and motoneurons and maintains their electrophysiological features. Both adult DRG sensory neurons and embryonic spinal motoneurons were able to adhere on 3 µm width lines. The presence of microplots with diameters varying from 10 to 50 µm not only did not promote a surface-based selection among the various neuronal subpopulations, but also failed to promote neuronal adhesion relative to lines, which is different from what was reported with other cell types [19], [30]. This suggests that the limiting factor for sensory and motoneuron adhesion is the probability to encounter an appropriate adhesive surface at the time of seeding.

The line micropattern used in the present study was designed for analysis of the neuronal electrical activity under a morphological constraint encountered for *in vivo* growth or regeneration of the mixed sensory-motor peripheral nerve. Such patterns have been shown to be useful for axon formation and guidance mechanisms [19], [20], [31], as well as to control alignment of Schwann cells and glial supportive cells, which provide guidance during development and regeneration [32]. Here, we show that micropattern-guided DRG sensory neurons grow one or two neurites without secondary branching and thus fulfill the role of a guidance tube. Immunostaining demonstrated that, similar to *in vivo* situation, the two emerging neurites were indeed axons, but not dendrites. In addition, we demonstrated a polarization of multipolar central neurons by our line micropatterning that definitively allows the axonal-dendritic polarization of motoneurons and confirms this cell-autonomous process. Importantly, we show that the line micropattern does not prevent the effects of conditioning induced by a prior *in vivo* peripheral nerve injury on neurite length of both the small and large population of DRG sensory neurons as reported on unpatterned substrate [4], [6]. However, the line pattern reveals subtle differences in neurite growth between the small and large size DRG neurons. While conditioning increases the number of neurons extending a neurite exclusively among the large population, it significantly increases length of the second neurite only among the small population of DRG neurons. Thus the line patterning allows demonstrating, for the first time, that neurite initiation and extension could be differentially regulated by conditioning injury among DRG sensory neuron subpopulations. However, with regards to the wide functional diversity of DRG sensory neurons, the neurite regrowth appears as a rather function-independent process. The functional significance of two neurites *in vitro* could be related to the peripheral and central axonal branches arising from each DRG sensory neurons *in vivo*. Interestingly, *in vivo*, peripheral conditioning was shown to promote not only regeneration of the peripheral branch, but also central branch of DRG sensory axons into the spinal cord [33], [34]. However the identity of DRG sensory neurons primed to grow into the spinal cord was not addressed. In the present study, we used laminin as a permissive substrate for both peripheral and central neurite growth of DRG sensory neurons and spinal motoneurons. Our model could also be applied to the nerve regeneration in the central nervous system after an injury. Following spinal cord trauma, chondroitin sulfate proteoglycans participate in the formation of the extracellular matrix and are major inhibitory components of the glial scar [35]. Using a patterned non-permissive substrate, it would be of interest to evaluate the effects of the conditioning paradigm on neurite initiation and extension together with electrical properties.

Lastly, our study shows that electrical activity of motoneurons and the large somatic size subpopulation of DRG sensory neurons is preserved on a line-patterned substrate. As we observed on unpatterned substrate, embryonic motoneurons at 2 DIV fire only small amplitude action potential. Full action potential amplitude and repetitive activity of mature motoneurons is recorded from 7 DIV, a time point that we did not evaluate on patterned substrate [36]. For patterned adult DRG sensory neurons, full action potential amplitudes were recorded, followed by two types of hyperpolarization: the fast AHP was a hallmark of conditioned neurons, while the slow AHP characterized control neurons. We previously recorded similar electrical expression profile on unpatterned substrate, which demonstrates that morphology does not affect the expression of these voltage-dependent K^+^ currents [8]. In addition, the subset of large DRG sensory neurons expressing an ADP was preserved [22]. Remarkably, patterning induced a significant decrease in the threshold current amplitude required to trigger an action potential in control neurons. The micro-patterned substrate impacts the excitability of sensory neurons and promotes the apparition of firing action potentials characteristic for a subclass of mechano-proprioceptors. The major effects of line pattern are to simplify cell geometry and to reduce cell contact with the substrate. While cell geometry could directly impact the threshold current, it cannot account for the increased firing properties of patterned DRG sensory neurons. We hypothesize that membrane tension linked to neurite number and attachment to the substrate could influence the expression or activation of mechanosensitive channels known to impact firing properties of DRG sensory neurons [37]. Regulation of gene expression through nuclear tension could also contribute to shaping electrical activity [38]. Therefore, force interactions linked to cell geometry could contribute greater than expected to the electrical characteristics in particular in mechanosensitive neurons [39].

In conclusion, a structural design composed with narrow line width pattern with ECM proteins does not impair the regenerative program of DRG sensory neurons and maintains sub-population diversity with regards to electrical activity. It recapitulates specificity in neuronal polarization between central motoneurons and peripheral sensory neurons and could be quite relevant for analysis of central and peripheral sensory neurites growth. It offers a unique model for the analysis of the impact of geometry and space constraint on expression and activity of mechanosensitive channels in DRG sensory neurons as well as in motoneurons for which few is known on their functional implication.

## Materials and Methods

### Photolithography and molding

Parallel lines three-fold wider than a neurite (3 µm width) and 5 mm long were designed with the Raith Elphy Quantum software. The designed patterns consisted in lines separated by a distance of 200 µm to avoid cross-talk between each other. In addition, each line contains in the middle a microplot of various, 10, 20, 30 or 40 µm diameters intended to favor adhesion of neuronal somas (Figure 1A). A chromium mask was built according to our plan (Optimask society). According to the protocol reported in [40], a cleaned silicon master that will be used later as a mold was prepared in our ATEMI (Atelier de Technologie Microélectronique at University Montpellier 2) laboratory facility by spin coating a thin layer (10 µm) of SU8 negative photoresist (SU8 2010, Microchem), then irradiated through the mask. The non-irradiated zones were dissolved in SU8 developer (Microchem). The master was finally silanized with (tridecafluoro 1,1,2,2,-tetrahydrooctyl) trichlorosilane for 2 h under vacuum. Poly-dimethylsiloxane (PDMS) was used as a stamping material. A mixture of Sylgard 184 silicon elastomer curing agent and of Sylgard 184 silicon elastomer base (Dow Corning, 1∶10 v/v) was poured, cured for 3 h at 200°C, and carefully peeled off from the master and shortly exposed to oxygen plasma cleaning.

### Microcontact printing

Microcontact printing was adapted from previously described methods [41]. The cell culture substratum was an extracellular matrix (ECM) gel (Sigma-Aldrich, St Louis, MO, USA) composed primarily of β1-laminin, collagen type IV, heparan sulfate proteoglycan and entactin. ECM was used as a printing ink. The PDMS stamps were wetted with ECM diluted at 1∶10 in Dulbecco’s Modified Eagle Medium (Sigma-Aldrich) for 30 minutes then washed twice in PBS. The stamps were dried with light nitrogen flow and printed on oxygen plasma cleaned polystyrene coverslips. The polystyrene substrates were then wetted with 0.1 mg/ml PLL-g-PEG solution (Surface and Solutions) in 10 mM HEPES pH 7.4 (Life Technologies, Carlsbad, CA, USA) for 1 h at room temperature. This step was necessary to render the non-printed areas resistant to cell adhesion and to enable cell confinement on lines as narrow as 3 µm. The substrate was finally rinsed twice with PBS to render it ready for cell deposition.

### Surgery and sensory neuron culture

Animals were housed in facilities accredited by the French ministry of agriculture and forestry (B-34 172 36–March 11, 2010). Adult Swiss female mice (6–10 weeks old, CERJ, Le Genest St. Isle, France) and male *Hb9::GFP* mice were housed in cages with a 12 h light/dark cycle and fed food and water *ad libitum.* The care and use of mice conformed to institutional policies and guidelines. Experimental procedures were approved by the local ethics committee and the protocols were validated by the Direction Départementale des Services Vétérinaires de l'Hérault (Certificate of Animal Experimentation No. B 34–65, 17 August 2010). For surgery, mice were deeply anesthetized by isoflurane inhalation. The left sciatic nerve was exposed at the mid-thigh, sectioned, and a 3–5 mm fragment was removed. Four days after surgery, mice were killed by CO_2_ inhalation followed by cervical dislocation and lumbar (L4–L5) dorsal root ganglia were removed. For each neuronal culture, contralateral (control) and ipsilateral (conditioned) L4–L5–L6 lumbar dorsal root ganglia from two operated mice were used. The cell cultures were established as previously reported [6]. Neurons were seeded at a density of 3,000 cells per cm^2^ patterned dishes in supplemented Neurobasal (Life Technologies) medium completed with 2% B27 and 2 mM L-glutamine (Life Technologies). The cells were maintained at 37°C in 5% CO2 atmosphere.

### Motoneuron culture

Motoneuron cultures were prepared from embryonic day (E)12.5 *Hb9::GFP* mice as described previously [42]. Briefly, ventral spinal cord was dissected and cut in small pieces. Cells were dissociated mechanically after trypsin treatment of ventral spinal cords. The largest cells were isolated using iodixanol density gradient purification. After counting green fluorescent neurons with a Malassez counting chamber, motoneurons were seeded at a density of 2,500 per 2 cm^2^ in Neurobasal medium (Life Technologies) supplemented with 2% (vol/vol) horse serum, 2% (vol/vol) B-27 supplement, 25 mM L-glutamate, 25 mM β-mercaptoethanol, 0.5 mM L-glutamine and a combination of neurotrophic factors (1 ng/ml BDNF, 100 pg/ml GDNF, and 10 ng/ml CNTF). To select the α-motoneurons, responsible for muscle contraction, experiments were conducted on *Hb9::GFP* neurons having a large somatic diameter (≥20 µm) [43].

### Immunofluorescence

Neuronal cultures were fixed for 15 min in 4% paraformaldehyde in phosphate-buffered saline (PBS), and incubated for 20 min in blocking buffer (10% donkey serum in 0.1% Triton-X100 in PBS). They were then incubated overnight at 4°C with the primary antibodies in the blocking buffer diluted to 1∶10. Cell cultures were then incubated for 1 h at room temperature with secondary antibody in the blocking buffer 1∶10, followed by mounting in Mowiol. For neurite analysis, images were obtained with a PL-Neofluar Zeiss 10×0.3 or 20×0.8 objectives mounted on an upright Zeiss microscope equipped with a CCD camera AxioCam MRm, using AxioVision software and quantified with ImageJ (V1.47a). Primary antibodies used were mouse anti-βIII-tubulin (Sigma Aldrich; 1∶400), rabbit anti-βIII-tubulin (Sigma-Aldrich; 1∶400), rabbit anti-laminin (Sigma-Aldrich; 1∶100), mouse anti Map2a/b (Sigma-Aldrich; 1∶1000), chicken anti-GFP (Abcam, Cambridge, MA, USA; 1∶3000), and mouse anti SMI312 (Abcam; 1∶1000). Secondary antibodies were Alexa Fluor-488, Alexa Fluor-594 or Alexa Fluor-647 (Life Technologies, 1∶500).

### Electrophysiological recordings

Electrophysiological recordings in dorsal root ganglion neurons were done at 2 days culture *in vitro* with the whole-cell patch-clamp technique. For action potential recordings the bathing solution contained: 140 mM NaCl, 5 mM KCl, 2 mM CaCl_2_, 1.5 mM MgCl_2_, 10 mM HEPES, 10 mM glucose and the pH was adjusted to 7.4 with NaOH. Recording pipettes were filled with the following solution: 145 mM KCl, 10 mM HEPES, 2 mM Mg-ATP, 0.5 mM Na_2_-GTP, 10 mM ethylene-glycol-bis(2-aminoethylether)-N,N,N’,N’-tetraacetic acid (EGTA) and pH7.35 adjusted with KOH. All recordings were made at room temperature using an Axopatch 200B amplifier (Dipsi Industrie, Chatillon, France). The experimental parameters were controlled by Digidata 1200 analogue interface (Axon Instrument). We used pClamp software (Clampex 8.02; Axon Instruments) for data acquisition and analysis. Signals were filtered at 2 kHz and sampled at 5 kHz, respectively. For each experiment, cell size was estimated by means of an eyepiece micrometer scale and only patterned neurons having somatic diameter superior to 30 µm were selected for these experiments.

### Statistical analyses

All values are reported as mean ± standard error of the mean (s.e.m). Statistical significance was evaluated using Student's *t* test or Chi-square (and Fisher’s exact) test as indicated. *p*<0.05 was considered as significant.

## Supporting Information

Figure S1 **SMI312 is a marker of large sensory neurons, not small neurons.** (red: anti-βIII tubulin for neuronal cytoskeleton; green: anti-SMI312 for axon). Only the neuron with large size soma is positive to SMI312.
(TIF)

Figure S2 As a dendrite marker, Map2a/b stains only short processes of motoneurons (at 6 DIV). (red: anti-Map2; green anti-GFP to enhance staining of the Hb9-GFP motoneurons).
(TIF)
