# Supplementary figures and images for: Morphology and Intrinsic Excitability of Regenerating Sensory and Motor Neurons Grown on a Line Micropattern
Source: PLoS One. 2014 Oct 20;9(10):e110687. doi: 10.1371/journal.pone.0110687 (PMC4203813; doi:10.1371/journal.pone.0110687)

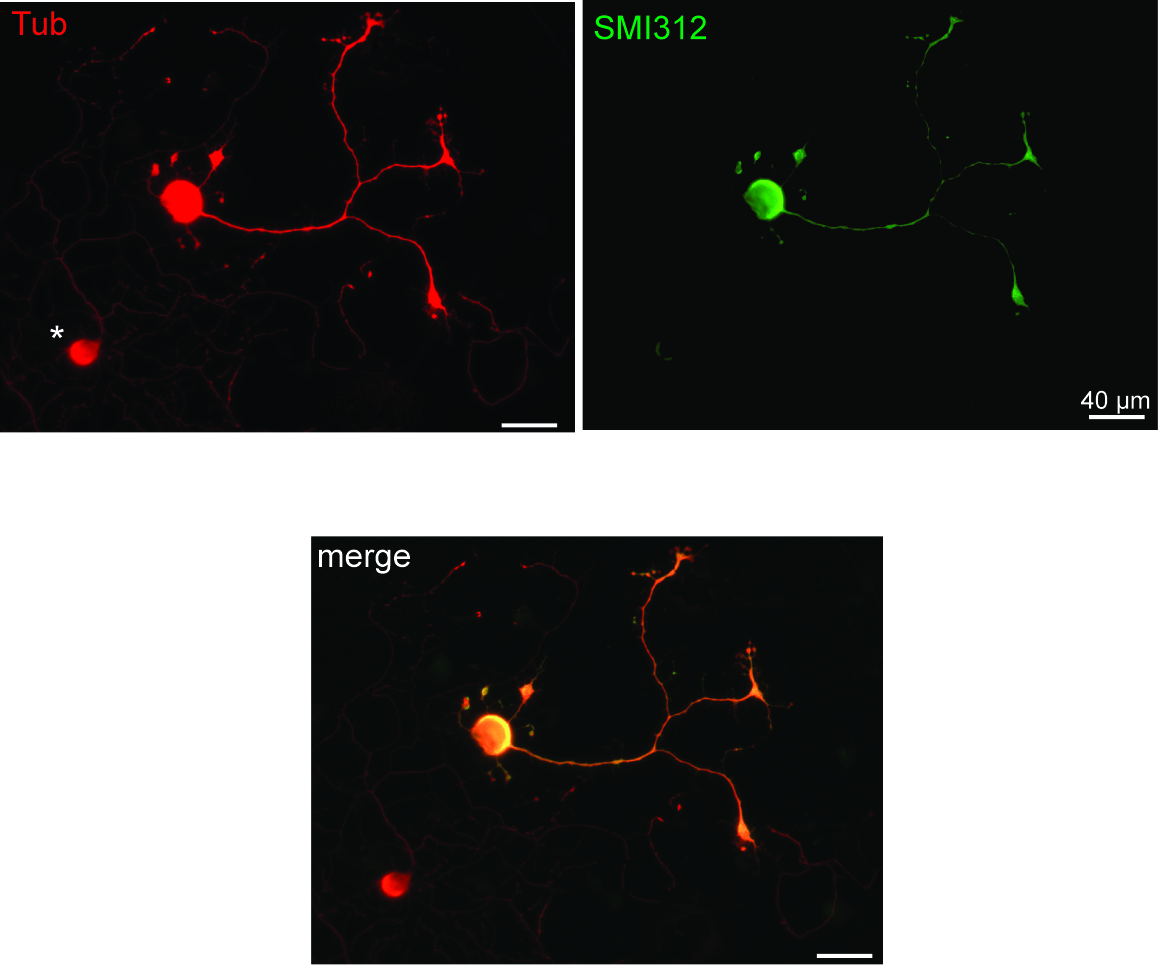

Supplement: Figure S1 — SMI312 is a marker of large sensory neurons, not small neurons. (red: anti-βIII tubulin for neuronal cytoskeleton; green: anti-SMI312 for axon). Only the neuron with large size soma is positive to SMI312. (TIF) [file pone.0110687.s001.tif]

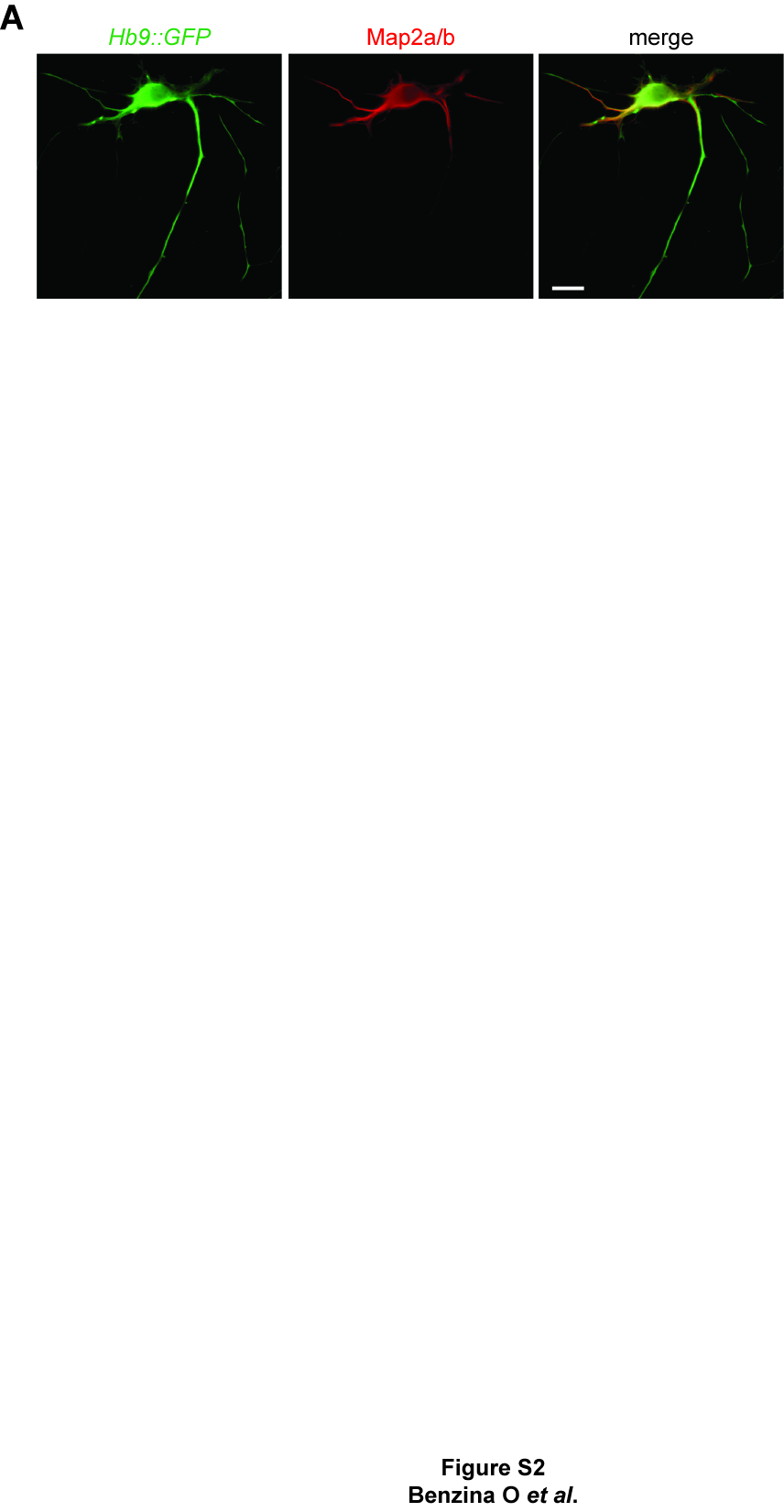

Supplement: Figure S2 — As a dendrite marker, Map2a/b stains only short processes of motoneurons (at 6 DIV). (red: anti-Map2; green anti-GFP to enhance staining of the Hb9-GFP motoneurons). (TIF) [file pone.0110687.s002.tif]
